# WHO CARES? RETENTION STRATEGIES FOR DIRECT CARE WORKERS

**DOI:** 10.1093/geroni/igad104.0974

**Published:** 2023-12-21

**Authors:** Robert Applebaum, Leah Janssen, Robert Applebaum

## Abstract

More than 30 years ago the Gerontological Society sponsored a congressional briefing about a growing problem; concerns about the adequacy and quality of the direct care workforce in long-term care. In 2002 the Robert Wood Johnson Foundation sponsored the Better Jobs, Better Care demonstration, in response to this continued challenge. Today recruiting and retaining high quality workers in the long-term services system has moved beyond a challenge to an all-out crisis. The initial paper of the symposium presents recent retention and work culture data highlighting todays’ hurdles, collected from more than 80% of long-term care facilities across Ohio. The second presentation provides a comprehensive review of state-level initiatives and innovations aimed at improving recruitment and retention for direct care workers (DCWs) in both facility-based long-term care and home and community-based long-term services across the country. A third paper focuses on retention-promoting factors from the perspective of providers and DCWs working in high-performing long-term care facilities in Ohio. In-depth interviews with 37 providers and DCWs revealed an overarching theme of the importance of work culture, highlighting the modifiable, intangible aspects for promoting DCW retention. The final paper introduces a tip sheet with practical strategies for providers who are seeking to retain direct care workers. Symposium findings will include both practical implications for long-term care providers and ideas for future studies. The direct care workforce crisis is the result of an array of challenges, which means that there will not be one solution, but rather many ideas will be needed.

